# Structures of the human Pals1 PDZ domain with and without ligand suggest gated access of Crb to the PDZ peptide-binding groove

**DOI:** 10.1107/S139900471402776X

**Published:** 2015-02-26

**Authors:** Marina E. Ivanova, Georgina C. Fletcher, Nicola O’Reilly, Andrew G. Purkiss, Barry J. Thompson, Neil Q. McDonald

**Affiliations:** a Structural Biology Laboratories, Cancer Research UK, 44 Lincoln’s Inn Fields, London WC2A 3LY, England; b Epithelial Biology Laboratories, Cancer Research UK, 44 Lincoln’s Inn Fields, London WC2A 3LY, England; c Peptide Chemistry Laboratories, Cancer Research UK, 44 Lincoln’s Inn Fields, London WC2A 3LY, England; d Institute of Structural and Molecular Biology, Department of Biological Sciences, Birkbeck College, University of London, Malet Street, London WC1E 7HX, England

**Keywords:** PDZ domains, Crb, cell polarity, epithelia, stardust

## Abstract

Many components of epithelial polarity protein complexes possess PDZ domains that are required for protein interaction and recruitment to the apical plasma membrane. Apical localization of the Crumbs (Crb) transmembrane protein requires a PDZ-mediated interaction with Pals1 (protein-associated with Lin7, Stardust, MPP5), a member of the p55 family of membrane-associated guanylate kinases (MAGUKs). This study describes the molecular interaction between the Crb carboxy-terminal motif (ERLI), which is required for *Drosophila* cell polarity, and the Pals1 PDZ domain using crystallography and fluorescence polarization. Only the last four Crb residues contribute to Pals1 PDZ-domain binding affinity, with specificity contributed by conserved charged interactions. Comparison of the Crb-bound Pals1 PDZ structure with an apo Pals1 structure reveals a key Phe side chain that gates access to the PDZ peptide-binding groove. Removal of this side chain enhances the binding affinity by more than fivefold, suggesting that access of Crb to Pals1 may be regulated by intradomain contacts or by protein–protein interaction.

## Abstract 2

This study characterizes the interaction between the carboxy-terminal (ERLI) motif of the essential polarity protein Crb and the Pals1/Stardust PDZ-domain protein. Structures of human Pals1 PDZ with and without a Crb peptide are described, explaining the highly conserved nature of the ERLI motif and revealing a sterically blocked peptide-binding groove in the absence of ligand.

## 1. Introduction   

Epithelial cell polarity is maintained by the asymmetric distribution of discrete protein complexes at either the apical or the baso-lateral membrane (Tepass, 2012 ▶). These two membrane domains are separated by tight junctions (TJs) in vertebrates or adherens junctions (AJs) in *Drosophila*. Two protein complexes called the Par complex and the Crb complex are implicated in apical polarity and contain components with multiple PDZ domains (Bilder *et al.*, 2003 ▶). The Par complex consists of the PDZ-domain proteins Par3 (partitioning defective 3 homologue) and Par6 (partitioning defective 6 homologue), aPKC (atypical protein kinase C) and Cdc42 (cell division control protein 42 homologue). The Crb complex contains the transmembrane protein Crumbs (Crb), the scaffold proteins MALS (mammalian Lin-7 isoforms 1, 2 and 3), the PDZ-domain protein Pals1 (protein associated with Lin-7) and PATJ (Pals1-associated TJ protein) (Tepass, 2012 ▶).

**Figure 1:**
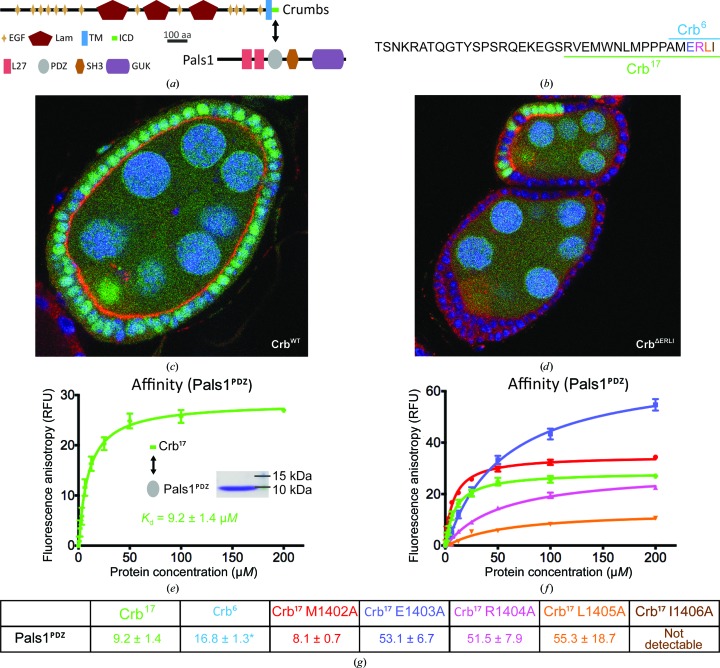
*In vivo* and *in vitro* interaction between Stardust/Pals1 and Crumbs/Crb1. (*a*) Schematic for Pals1 and Crumbs domain organization. EGF, epidermal growth factor; LamAG, laminin AG-like; TM, transmembrane; ICD, intracellular; L27, lin-2, lin-7 homology; PDZ, postsynaptic density 95, disc-large, zona occludens homology; SH3, sarcoma homology 3; GUK, guanylate kinase. The PDZ domain of Pals1 interacts with Crumbs through its ICD. (*b*) The amino-acid sequence of the Crb^17^ peptide used for biochemical experiments and for crystallization is underlined; the PDZ-binding motif is coloured. (*c*) *D. melanogaster* Crumbs (shown in red) localizes to the apical membrane of the follicle cells of egg chambers. Wild-type follicle cells (WT) are marked by the presence of green fluorescent protein (GFP). (*d*) Deletion of the four C-terminal residues of Crumbs (Crb^ΔERLI^) causes a failure of Crb to localize to the apical plasma membrane of the follicle cells and disrupts polarity. Mutant cells are marked by the absence of GFP; some GFP-positive cells can be seen. (*e*) Representative binding curves for fluorescein-labelled Crb^17^ for Pals1^PDZ^ and (*f*) Crb^17^ mutants measured by fluorescence polarization (Crb^17^, green; Crb^17^ L1405A, orange; Crb^17^ R1404A, pink; Crb^17^ E1403A, blue; Crb^17^ M1402A, red). The affinity between Crb^17^ I1406A and Pals1^PDZ^ could not be reliably determined. (*g*) Summary of dissociation constants (in µ*M*) between Crb^17^ and Pals1^PDZ^ [colour-coded as in (*f*); Crb^17^ II406A, brown, Crb^6^, light blue] measured by Fp with fluorescein-labelled peptides. All values shown were measured in parallel from the same Pals1 preparation (except for the Crb^6^ peptide, which was measured using a different Pals1^PDZ^ preparation; the *K*_d_ between Crb^17^ and Pals1^PDZ^ from this preparation was 12.4 ± 2.4 µ*M*). These data were representative of three independent experiments.

Crb consists of a large extracellular domain (ECD), a transmembrane region (TM) and a 37-amino-acid intracellular domain (ICD). It has been shown that the Crb ECD (Crb^ECD^) can oligomerize to mediate cell adhesion in the retina (Zhou & Hong, 2012 ▶; Fletcher *et al.*, 2012 ▶). The Crb^ICD^ contains two protein-binding motifs: a juxtamembrane FERM (band 4.1, ezrin, radixin, moesin)-binding motif (FBM) and a carboxy-terminal PDZ-binding motif. Crb recruits Pals1 to the apical membrane *via* an interaction between Crb^ICD^ and Pals1^PDZ^, and this interaction is required for Crb localization at the apical membrane (Bachmann *et al.*, 2001 ▶; Fig. 1 ▶ *a*). Pals1 belongs to the MPP family of proteins (membrane protein, palmitoylated), also known as the p55 subfamily of membrane-associated guanylate kinases (MAGUKs). Pals1 acts as a scaffold protein, recruiting other proteins to the apical domain of the cells: the L27 domains of Pals1 bind to Patj and Lin7, while the amino-terminal regions of Pals1 can interact with the PDZ domain of Par6, linking the Crb and Par complexes (Hurd *et al.*, 2003 ▶). It has also been shown that the PDZ domain of Par6 can bind directly to the PDZ-binding motif (PBM) of Crb (Hurd *et al.*, 2003 ▶; Kempkens *et al.*, 2006 ▶).

PDZ domains form a large diverse family that were first described to selectively recognize carboxy-terminal peptides (PDZ-binding motifs or PBMs) of their target proteins (Subbaiah *et al.*, 2011 ▶). The α/β architecture of the PDZ domain defines a peptide-binding groove for the PBM and a key carboxylate-binding loop. PDZ domains are usually grouped into three classes based on the C-terminal PBM sequence that they recognize. These include type I PBMs (-*X*-S/T-*X*-Φ_COOH_), type II PBMs (-*X*-Φ-*X*-Φ_COOH_) and type III PBMs (-*X*-D/E-*X*-Φ_COOH_), where *X* is any amino acid and Φ is any hydrophobic amino acid (Harris & Lim, 2001 ▶; Songyang *et al.*, 1997 ▶). Recent studies have shown that adjacent regions to the peptide-binding groove of PDZ domains or even residues in domains flanking the PDZ domain can contribute towards the PBM binding the protein (Zhang, Dasgupta *et al.*, 2007 ▶; Bhattacharya *et al.*, 2013 ▶; Nomme *et al.*, 2011 ▶; Pan *et al.*, 2011 ▶). PDZ domains can also bind to internal PBMs (Lenfant *et al.*, 2010 ▶; Penkert *et al.*, 2004 ▶; Zhang, Appleton *et al.*, 2007 ▶), other PDZ domains (van den Berk *et al.*, 2007 ▶; Chang *et al.*, 2011 ▶), phosphatidylinositol 4,5-bisphosphate [PIP(2); Zimmermann *et al.*, 2002 ▶] or even lipids (Chen *et al.*, 2012 ▶; Zimmermann *et al.*, 2002 ▶). Several mechanisms have been described to regulate the PDZ–PBM interaction. Phosphorylation of the PBM can increase affinity for the PDZ domain (Tyler *et al.*, 2010 ▶), whilst phosphorylation of the carboxylate-binding loop can abolish PBM interaction altogether (Raghuram *et al.*, 2003 ▶). Protein partners can switch PDZ ligand-recognition modes, for example, Cdc42 binding to Par6 switches its PDZ from binding an internal Pals1 peptide sequence to a carboxyl-terminal motif (Penkert *et al.*, 2004 ▶; Whitney *et al.*, 2011 ▶; Peterson *et al.*, 2004 ▶).

This study characterizes the carboxy-terminal (ERLI) motif of Crb, which is crucial *in vivo* for polarity and binds to Pals1/Stardust. The crystal structure of a human Pals1^PDZ^–Crb^PBM^ complex is described that explains the highly conserved nature of the ERLI motif and details the contacts. Biophysical characterization supports a essential role for just the four C-terminal residues. The structure of ligand-free Pals1^PDZ^ reveals a sterically blocked peptide-binding groove, as confirmed by fluorescence polarization *in vitro*. Comparisons of liganded and apo Pals1^PDZ^ reveals conformational rearrangements upon binding the Crb ERLI motif and suggest regulated access to the Pals1 PDZ peptide-binding pocket.

## 2. Experimental procedures   

### 2.1. Fly strains and generation of clones   

Mitotic clones were generated using the Flp-*FRT* method of recombination. Third-instar larvae of the following genotype were heat-shocked at 37°C for 1 h: *hsflp;; frt82B crb^ΔPBM^/frt82B ubiGFPnls* (a kind gift from D. J. Pan).

### 2.2. Antibodies and immunohistochemistry   

Ovaries were dissected in PBS, fixed for 20 min in 4% PFA, washed for 30 min in PBS/0.1% Triton X-100 (PBST) and blocked for 15 min in 5% normal goat serum/PBST (PBST/NGS). The primary antibody was diluted in PBST/NGS and samples were incubated overnight at 4°C. We used rat anti-Crumbs (1:200; a kind gift from E. Knust). Secondary antibodies were used at 1:500 and DAPI at 1 µg ml^−1^ (all from Molecular Probes, Invitrogen). Images were taken with a Leica SP5 confocal microscope.

### 2.3. Protein-construct design, expression and purification   

Plasmids encoding cDNAs for the human Pals1 PDZ domain (wild-type and F318A mutant) were transformed into *Escherichia coli* FB810 cells and grown in LB medium at 37°C in the presence of anitibiotics. After reaching a density of *A*_600_ = 0.6, the cells were induced with 20 µ*M* IPTG (Sigma–Aldrich) and grown at 16°C for 18 h with agitation. The cells were harvested and resuspended in 20 m*M* HEPES pH 7.5 (Sigma), 100 m*M* NaCl (Sigma), 10 m*M* Benzamidine, 0.2 m*M* AEBSF, 1 m*M* DTT. The cells were disrupted by sonication and spun down at 30 000*g* for 30 min. Pals1^PDZ^ protein was extracted from the lysate using glutathione Sepharose 4B beads (Amersham Biosciences) and washed in 20 m*M* HEPES pH 7.5, 100 m*M* NaCl, 1 m*M* DTT, followed by removal of the GST affinity tag with GST-3C protease (PreScission Protease, Amersham Bioscience) overnight at 4°C. The eluate was further purified by size-exclusion chromatography (Superdex S75). All purification steps were performed at 4°C or on ice. Protein purity was analysed using SDS–PAGE.

### 2.4. Fluorescence polarization assays to determine the dissociation constants (*K*_d_)   

Fluorescence polarization (Fp) assays were performed to determine the *K*_d_ for each peptide following a previously described protocol (Guettler *et al.*, 2008 ▶). Binding assays were performed in 20 m*M* HEPES pH 7.5, 100 m*M* NaCl, 1 m*M* DTT. The reaction mixtures contained a fixed concentration of fluorescein-labelled peptide (50 n*M*) and a protein concentration ranging from 0 to 300 µ*M* depending on the dissociation constant. The 20 µl reactions were carried out in a 384-well plate and measured after 5 min using a Tecan Safire2 plate reader with excitation at 470 nm and emission at 525 nm. The anisotropy values were normalized and the *K*_d_ values were determined using nonlinear regression with the graphics program *Prism* (Heyduk & Lee, 1990 ▶).

### 2.5. Structure determination of ligand-free Pals1^PDZ^ and Pals1^PDZ^ bound to Crb1 residues 1390–1406 (Crb^17^) peptide   

Pals1^PDZ^ was incubated with a two-molar excess of human Crumbs peptide (homologue 1; residues 1390–1406, defined hereafter as Crb^17^; RVEMWNLMPPPAMERLI) for 30 min on ice. Crystals were grown at 20°C by vapour diffusion in sitting drops consisting of 0.15 µl protein stock solution (5 mg ml^−1^) mixed with 0.1 µl reservoir solution (0.1 *M* HEPES pH 7.29, 2.68 *M* NaCl). These crystals grew to maximum size in 4 d. Crystals were cryoprotected in 50% Paratone, flash-cooled in liquid nitrogen and an X-ray data set was collected on the I04-1 beamline at Diamond Light Source, Oxford, England. The data set was indexed and scaled using *xia*2 (Winter *et al.*, 2013 ▶). The crystal belonged to space group *P*4_1_22. Data-processing and refinement statistics are presented in Table 1 ▶. Molecular replacement was carried out with *Phaser* (McCoy *et al.*, 2007 ▶) using the model generated by the *Phyre*2 server (Kelley & Sternberg, 2009 ▶) based on sequence alignment of Pals1–PDZ against all structures in the PDB. One copy of Pals1^PDZ^ bound to one copy of Crb^17^ was found in the asymmetric unit. Difference electron density corresponding to the Crb^17^ peptide was clearly visible after molecular replacement and after initial rounds of refinement. The structure was refined using *PHENIX* (Adams *et al.*, 2010 ▶), with model building carried out in *Coot* (Emsley *et al.*, 2010 ▶). The Crb^17^ peptide was built into electron density (with residues 1390–1392 disordered); Crb^17^ Met1402 was oxidized, most likely owing to radiation damage during data collection. The vector-derived residues Gly-Pro-Leu-Gly-Ser at the amino-terminus of Pals1^PDZ^ had electron density and so were included in the refined model.

Crystals of the ligand-free form of Pals1^PDZ^ were grown at 4°C by vapour diffusion in sitting drops consisting of 0.1 µl protein stock solution (6.5 mg ml^−1^) mixed with 0.15 µl reservoir solution (0.085 *M* Tris pH 8, 0.17 *M* sodium acetate, 19% glycerol, 25.9% PEG 4000). These crystals grew to maximum size in 24 h. The crystals were flash-cooled in liquid nitrogen without additional cryoprotection and an X-ray data set was collected on the I04-1 beamline at Diamond Light Source, Oxford, England. The data set was indexed and scaled using *xia*2 (Winter *et al.*, 2013 ▶). The crystals belonged to space group *P*4_3_2_1_2. Molecular replacement was carried out in *Phaser* (McCoy *et al.*, 2007 ▶) using the atomic coordinates of the previously solved PDZ domain of Pals1 in complex with the Crb^17^ peptide as a search model. One copy of Pals1^PDZ^ was found in the asymmetric unit. The structure was refined using *PHENIX* (Adams *et al.*, 2010 ▶) and model building was carried out in *Coot* (Emsley *et al.*, 2010 ▶). The coordinates and structure factors have been deposited in the Protein Data Bank with accession codes 4uu5 (Pals1^PDZ^–Crb^17^ complex) and 4uu6 (ligand-free Pals1^PDZ^). Figures were prepared using the graphics program *PyMOL* (http:/www.pymol.org).

## 3. Results   

### 3.1. Apical localization of Crb requires Stardust interaction and is essential for polarity *in vivo*   

Crumbs (Crb) is a large type I single membrane-spanning protein containing a short intracellular domain whose carboxyl-terminal residues ERLI can bind the PDZ domain of Stardust (the human orthologue is Pals1; Figs. 1 ▶ *a* and 1 ▶ *b*). Crb is localized to the apical membrane of epithelial cells such as the follicle cells of *D. melanogaster* egg chambers (Fig. 1 ▶ *c*). This apical localization is dependent on its interaction with Stardust, as deleting the PDZ-binding ERLI motif disrupts Crb localization and polarity in a follicle-cells context (Fig. 1 ▶ *d*).

### 3.2. Characterization of Pals1^PDZ^–Crb^17^ interaction by fluorescence polarization   

To characterize the interaction between the PDZ domain of human Pals1 (Pals1^PDZ^; residues 251–335) and the intra­cellular segment of human Crb1 *in vitro*, we used fluorescence polarization (Fp) assays with a fluorescein-labelled peptide containing 17 carboxy-terminal residues 1390–1406 (defined hereafter as Crb^17^). A high-affinity site for Crb^17^ was measured for Pals1^PDZ^ with a dissociation constant (*K*_d_) of 9.2 ± 1.4 µ*M* (Figs. 1 ▶ *e* and 1 ▶ *f*). The Pals1^PDZ^–Crb^17^ interaction was stable to size-exclusion chromatography as detected from the Crb^17^ intrinsic tryptophan fluorescence (data not shown). Alanine substitutions within the Crb^17^ ERLI motif resulted in a substantial weakening of the affinity for Pals1^PDZ^. Individual Crb^17^ substitutions E1403A, R1404A and L1405A gave similar detrimental impacts on *K*_d_, reducing it to between 52 and 55 µ*M*, independent of their position relative to the carboxy-terminus (Figs. 1 ▶ *e* and 1 ▶ *g*). Replacing the carboxy-terminal Ile1406 with alanine (I1406A) completely abolished detectable binding to Pals1^PDZ^. A shorter Crb1 peptide spanning residues 1401–1406 (defined hereafter as Crb^6^) showed an equivalent affinity for Pals1^PDZ^ as Crb^17^ and a Crb^17^ M1402A mutant. These data demonstrate that the Pals1^PDZ^ interaction is driven through the ERLI motif of Crb^17^, in particular the carboxy-terminal residue. This is consistent with the *in vivo* data showing the importance of the ERLI motif for the Pals1 interaction, as shown here and as described previously (Roh *et al.*, 2002 ▶; Makarova *et al.*, 2003 ▶; Bachmann *et al.*, 2001 ▶).

### 3.3. The Pals1^PDZ^–Crb^17^ structure rationalizes ERLI motif conservation   

**Figure 2:**
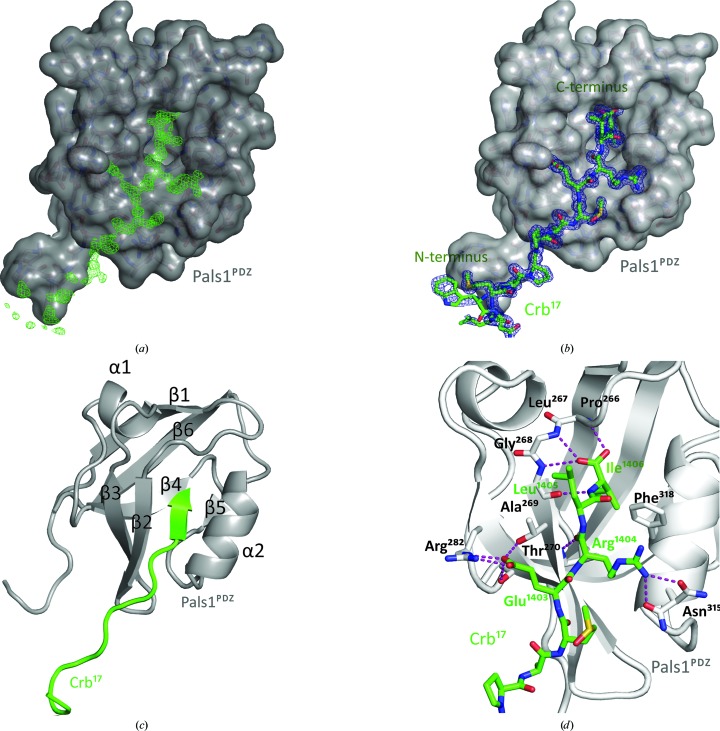
Overall structure and molecular interactions between Pals1^PDZ^ and Crb^17^. (*a*) Difference electron density (green) for the Crb^17^ peptide bound to Pals1^PDZ^ prior to fitting. The map is a σ_A_-weighted *F*_o_ − *F*_c_ electron-density OMIT map (contoured at σ = 3.0). (*b*) Electron density for the Crb^17^ peptide bound to Pals1^PDZ^ after refinement. The map is shown as a σ_A_-weighted 2*F*_o_ − *F*_c_ electron-density OMIT map (contoured at σ = 1.0). (*c*) Schematic representation of Crb^17^ (green) bound to Pals1^PDZ^ (grey cartoon). Secondary-structure elements are labelled starting from the N-terminus of Pals1^PDZ^. α, α-Helix; β, β-­strand. (*d*) Close-up of the Crb^17^ peptide (green sticks) bound to Pals1^PDZ^ (grey cartoon). Pals1 residues involved in the recognition of Crumbs are shown as sticks. Pink dashed lines represent hydrogen bonds that are established between the Crb^17^ peptide and Pals1^PDZ^.

To define the molecular interaction between human Pals1^PDZ^ and Crb^17^, we successfully crystallized the complex and determined its structure at 1.23 Å resolution. Data-processing and refinement statistics for the structure are presented in Table 1 ▶. Electron density for residues 1393–1406 of Crb^17^ was observed, allowing the fitting of all but three residues of the Crb^17^ peptide (Figs. 2 ▶ *a* and 2 ▶ *b*). A single Pals1^PDZ^–Crb^17^ complex is present within the asymmetric unit. The Pals1^PDZ^ adopts a canonical PDZ fold consisting of two β-sheets flanked by two α-helices. The carboxyl-terminus of the Crb^17^ peptide binds as an antiparallel β-strand extending the smaller PDZ β-sheet between strand β2 and helix α2 (Fig. 2 ▶ *c*). The total buried surface area between Pals1^PDZ^ and Crb^17^ is ∼500 Å^2^. The carboxy-terminal carboxylate group of Ile1406 in Crb^17^ forms hydrogen bonds to main-chain amides from the invariant Pals1 residues Leu267, Gly268 and Ala269 that define the canonical carboxylate-binding loop between strands β1 and β2 of Pals1^PDZ^ (Fig. 2 ▶ *d*). Leu321 of Pals1 adopts a strained side-chain rotamer on binding the ligand, allowing the side chain of Ile1406 from Crb^17^ to point into a deep hydrophobic pocket in Pals1^PDZ^. Van der Waals contacts between Crb^17^ Leu1405 and Pals1 Pro266 stabilize a closed carboxylate-binding loop enveloping the peptide ligand. Charged interactions with the ERLI motif are also found; Crb^17^ Glu1403 forms a salt bridge with Pals1^PDZ^ Arg282 and also forms hydrogen bonds to the side chains of Thr270 and Ser281. The Crb^17^ Arg1404 side chain makes a cation–π interaction with Phe318 and also forms hydrogen bonds to the Asn315 side chain (Fig. 2 ▶ *d*). Overall, the structure explains the tight conservation of each side chain of the Crb ERLI motif through selective interactions with the Pals1^PDZ^ domain, consistent with the binding affinities measured in solution by fluorescence polarization.

### 3.4. Structure of the ligand-free PDZ domain of Pals1   

**Figure 3:**
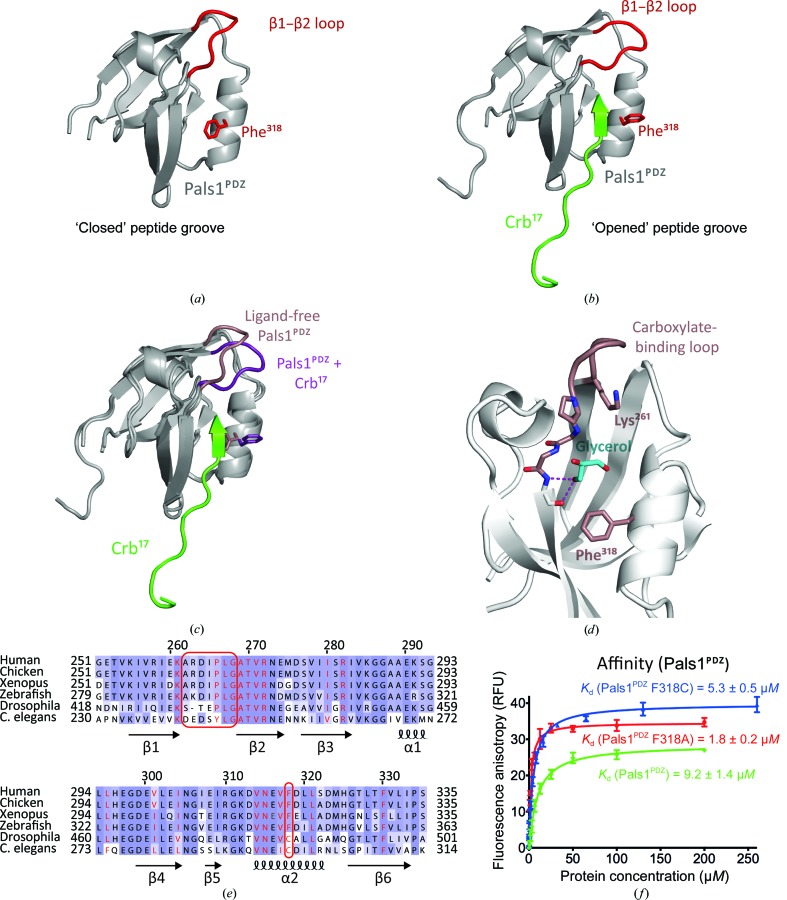
Comparison of ligand-free Pals1^PDZ^ and the Crb^17^–Pals1^PDZ^ complex. (*a*, *b*) Cartoon schematics of (*a*) ligand-free Pals1^PDZ^ and (*b*) the Crb^17^–Pals1^PDZ^ complex. (*c*) Superimposition of ligand-free Pals1^PDZ^ and the Crb^17^–Pals1^PDZ^ complex. Pals1^PDZ^ is shown in grey; Pals1 Phe318 and the carboxylate-binding loop are shown in red and the Crb^17^ peptide is shown in green. Elements with different conformers are highlighted in pink for unliganded Pals1^PDZ^ and in purple for the Crb^17^–Pals1^PDZ^ complex. (*d*) Close-up of the binding pocket of Pals1^PDZ^ (light grey) in the absence of peptide. The key structural elements which change conformation upon binding PBM are highlighted in pink. A glycerol molecule (shown in blue) occupies the PBM pocket, closely mimicking the carboxylate position of Crb^17^. (*e*) Sequence alignment of Pals1 isoforms across vertebrates and invertebrates. Conserved residues are highlighted in purple; darker purple indicates the most conserved residues. Residue numberings and secondary structures for human Pals1 are shown above and below the alignment, respectively; arrows represent β-strands and helices represent α-helices. Pals1 Phe318 and the carboxylate-binding loop are highlighted with red boxes and residues making contacts with the Crb^17^ peptide are highlighted in red. (*f*) Representative binding curves for a fluorescein-labelled Crb^17^ peptide binding to wild-type Pals1^PDZ^ (shown in green), Pals1^PDZ^ F318A mutant (shown in red) and Pals1^PDZ^ F318C mutant (shown in blue) measured by fluorescence polarization.

In order to investigate the conformational changes that occur in Pals1^PDZ^ upon binding Crb^17^ peptide, a ligand-free form of Pals1^PDZ^ was crystallized and its structure was determined at 1.8 Å resolution. This crystal form has a single copy of Pals1^PDZ^ in the asymmetric unit. Data-processing and refinement statistics for the structure are presented in Table 1 ▶. The ligand-free structure of Pals1^PDZ^ closely resembles the ligand-bound form, with an r.m.s. deviation of 0.607 Å over 60 C^α^ atoms, but with several important differences (Figs. 3 ▶ *a*, 3 ▶ *b* and 3 ▶ *c*). Firstly, the carboxylate-binding β1–β2 loop (residues 260–268) adopts a more open configuration pivoting at Glu260 and Leu267, resulting in a C^α^ shift of over 6.2 Å away from the C-terminal end of helix α2. This exposes the side chain of Pals1 Lys261 and ensures that this part of the PBM-binding groove is solvent-accessible. A glycerol molecule occupies the PBM pocket, closely mimicking the carboxylate position of Crb^17^ (Fig. 3 ▶ *d*). The Leu321 side chain adopts a favoured rotamer in the absence of a partner peptide. Another notable difference between ligand-free and ligand-bound Pals1^PDZ^ is the side-chain rotamer of Phe318 from helix α2, as discussed below (Fig. 3 ▶ *c*).

### 3.5. A gating mechanism for accessing the Pals1^PDZ^ peptide groove   

The Phe318 side-chain rotamer adopted in the apo Pals1^PDZ^ results in a steric block to the central part of the PDZ peptide-binding groove. In contrast, in the presence of the Crb^17^ peptide Phe318 swings out of the pocket to pack against the Arg1404 side chain of Crb^17^, as described earlier. Comparison with the previously deposited NMR structure of ligand-free Pals1^PDZ^ (PDB entry 1va8; RIKEN Structural Genomics/Proteomics Initiative, unpublished work) shows that in solution Phe318 also adopts the same rotamer as in the ligand-free X-ray structure, indicating that this is not a crystal lattice artefact (Supplementary Fig. S1*a*). From sequence comparisons, Pals1 Phe318 is conserved throughout vertebrate Pals1 sequences but is substituted by cysteine in invertebrate species (Fig. 3 ▶ *e*). Searching the Protein Data Bank (PDB) with *PDBeFold* (http://www.ebi.ac.uk/msd-srv/ssm/) against all PDZ-domain structures indicates that a phenylalanine at this position is unique to Pals1. We therefore considered whether Phe318 could act as a potential gating mechanism restricting access of ligand to the PDZ peptide groove. In this case, removing the Phe318 side chain by substituting it with alanine should increase the affinity between Pals1 and Crb^17^ by removing the steric block. This was indeed the case, as a Pals1^PDZ^ F318A mutation bound the Crb^17^ peptide fivefold more tightly than wild-type Pals1^PDZ^ domain (Fig. 3 ▶ *f*). Stardust/Pals1 homologues present in invertebrates have a cysteine at the equivalent position to Phe318. We therefore substituted Phe318 by cysteine and measured the effect on Crb interaction. This showed an intermediate affinity (5 µ*M*) for the Crb^17^ peptide between that of Phe318 and an alanine substitution. This suggests that gating of the PDZ ligand pocket by Phe318 is a property of vertebrate Pals1 proteins.

### 3.6. Comparison of the Pals1^PDZ^ and Par6^PDZ^ structures defines the basis for Crb^17^ recognition   

**Figure 4:**
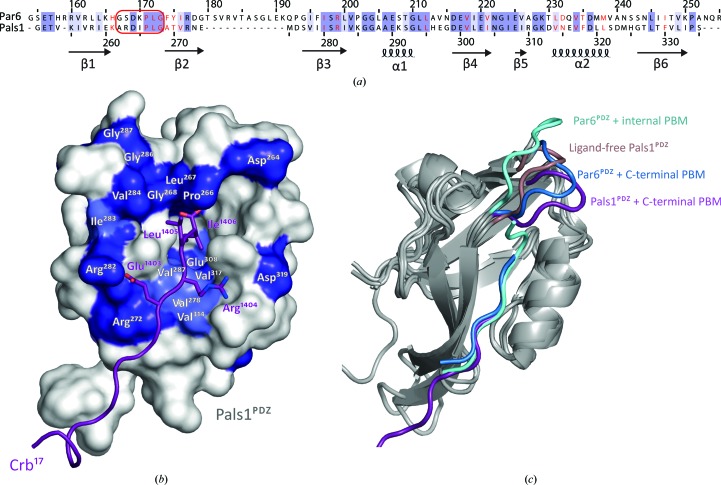
Crb^17^ recognition by Pals1^PDZ^ and Par6^PDZ^ and the similarity of ligand-free Pals1^PDZ^ to Par6^PDZ^ bound to an internal peptide ligand. (*a*) Sequence alignment of the human Pals1 and Par6 PDZ domains. Residue numbering for Par6 and Pals1 is shown above and below the alignment; secondary structure for Pals1 is shown below the alignment (arrows represent β-strands and helices represent α-helices); the carboxylate-binding loop is highlighted by a red box and residues making contacts with the Crb^17^ peptide are highlighted in red. (*b*) Surface conservation of Pals1/Par6 close to the ERLI ligand is shown in deep blue (identical), light blue (similar) and white (not conserved). The Crb^17^ peptide is shown in purple with the ERLI motif side chains, except for Arg1404, all making conserved contacts. This is consistent with the known Par6 PDZ-binding ligands ESLV (PDB entry 1rzx) and EMAV (PDB entry 1x8s), varying at the second position (equivalent to Arg1404 of Crb). (*c*) Superimposition of Par6^PDZ^ bound to the Pals1 internal ligand (light blue; PDB entry 1x8s), the ligand-free form of Pals1^PDZ^ (light pink), Par6^PDZ^ bound to the C-terminal ligand (dark blue; PDB entry 1rzx) and Pals1^PDZ^ bound to the C-terminal Crb^17^ ligand (purple). The carboxylate-binding loop in each structure is highlighted according to the colour legend in the figure.

The Par6 PDZ domain has also been shown to bind the Crb^ICD^ peptide (Hurd *et al.*, 2003 ▶; Kempkens *et al.*, 2006 ▶). In order to characterize this interaction, we compared sequence and structural alignments of the Pals1^PDZ^ and the Par6^PDZ^ to identify critical residues in common between both PDZ domains involved in Crb^17^ peptide recognition (Figs. 4 ▶ *a*, 4 ▶ *b* and 4 ▶ *c*). A number of Par6^PDZ^ ligands have been identified apart from the ERLI C-terminal Crb motif. Those ligands include the ESLV C-terminal motif (PDB entry 1rzx; Peterson *et al.*, 2004 ▶) and the Pals1 EMAV internal motif (PDB entry 1x8s; Penkert *et al.*, 2004 ▶); therefore, we can conclude that the Par6^PDZ^ binds to the C-terminal EXΦΦ motif or the EXAΦ internal motif. Superposition of the Par6^PDZ^ structure bound to a C-terminal peptide with the Pals1^PDZ^–Crb^17^ structure gave an r.m.s. deviation of 1.315 Å over 63 C^α^ atoms (Fig. 4 ▶ *c*). Both PDZ domains have an arginine side chain (Pals1 Arg282 and Par6 Arg199) to engage the glutamate of the Crb ERLI motif. Many apolar residues are conserved in the hydrophobic pocket that accommodates the carboxy-terminal side chain of Crb^17^ Ile1406 (see Fig. 4 ▶ *b*). No equivalent contacts from Pals1 or Par6 are made with the Arg1404 side chain of Crb, consistent with Par6^PDZ^ tolerating different side chains at this PDZ-ligand position. Residues Leu231 and Asp232 of Par6 are structurally equivalent to Val314 and Asn315 of Pals1 to engage this position of a PDZ ligand. Searching for this sequence signature through the human PDZome showed that only Par6 and members of the MPP (membrane protein, palmitoylated) family (MPP1, MPP3 and MPP5, also known as Pals1) have Arg, Asp/Asn and Leu/Val at these positions and therefore are potentially able to bind the Crb ERLI motif.

## 4. Discussion   

The interaction of Crb with Stardust/Pals1 is conserved from humans to flies. We show that deleting the PDZ-binding ERLI motif from fly Crb results in disrupted Crb polarization in follicle cells of *D. melanogaster* egg chambers. Similarly, disruption of Crb1 localization by a somatic heterozygous mutation E1403Q was found in a retinitis pigmentosa syndrome when combined with a C1154S substitution (Yang *et al.*, 2014 ▶). The invariant ERLI motif present in Crb deviates from a canonical PDZ-binding motif, requiring structural data to define the exact nature of this interaction. Our structure of a Crb^17^ peptide ligand bound to the Pals1 PDZ domain explains the basis for this conservation and presents detailed charged side-chain interactions, carboxylate-binding loop contacts and hydrophobic pockets for the penultimate two Crb residues.

Regulation of PDZ ligand binding is of considerable interest given the presence of single or multiple PDZ domains in many polarity proteins (Bilder *et al.*, 2003 ▶). Many have been presumed to exclusively bind canonical PDZ-binding motifs within the C-terminal tails of target proteins with micromolar affinity. However, there is increasing evidence for PDZ–PDZ domain interactions and that PDZ domains can frequently bind internal peptide ligands, including, for example, the Par6^PDZ^ and Pals1 internal ligand (Penkert *et al.*, 2004 ▶). We note that the ligand-free Pals1 PDZ domain closely resembles that of Par6, which is known to bind both internal and C-terminal peptide ligands. This is not surprising given that they both bind Crb *in vitro*. Examination of the open carboxylate-binding loop of ligand-free Pals1 PDZ domain with Par6 bound to an internal peptide ligand suggests that Pals1 could also bind internal ligands. This is supported by evidence from genome-wide yeast two-hybrid screening for partners of the *Caenorhabditis elegans* Pals1 homologue tag-117 (Lenfant *et al.*, 2010 ▶). This study identified many partners of tag-117 that bound independently of their C-terminal sequences, suggesting that tag-117 is able to recognize internal (noncanonical) peptide ligands in many cases. Interestingly, inspection of a superposition of the Pals1 PDZ domain with an internal peptide ligand for Par6 PDZ suggested that an E/D-*X*-small-Φ motif would permit the ligand main chain to exit the peptide groove with close contacts near the small side chain (Gly/Ser/Thr/Ala). Such a motif may define an appropriate sequence signature competent to bind Par6 and Pals1. Sequence searches with an E/D-(not P)-(SATGC)-(LIVMFA)-E/D-(SATGC)-P motif identified hits including the PDZ proteins MAGIX and MPDZ; the latter is a known partner of Pals1. This suggests future experiments to explore and confirm this prediction.

Our structural and biophysical data suggest steric regulation of access to the peptide-binding groove of the human Pals1 PDZ domain. We speculate that intradomain interactions outside the Pals1 PDZ domain may influence the Phe318 side-chain rotamer or, alternatively, binding of known partners to Pals could alter the side-chain conformer, triggering a conformation change within Pals1. There are many precedents for gated access to ligand pockets by bulky side chains such as phenylalanine (Koch *et al.*, 2011 ▶; Dostál *et al.*, 2005 ▶; Kaya *et al.*, 2014 ▶). However, to our knowledge this is the first example of gated access to a PDZ-domain peptide-binding groove. Future experiments will elaborate the structural and functional consequences of Crb^17^ peptide binding to full-length Pals1.

*Note added in proof.* While this article was under review, Li *et al.* (2014 ▶) published the structure of the same portion of the Crumbs C-tail bound to a larger Pals1 fragment containing PDZ-Src homology 3 (SH3)-guanylate kinase (GK) tandem domains. They report a similar affinity of the PDZ domain for Crumbs C-tail as our study. They also observed Crumbs C-tail contacts with Phe318 from the PDZ domain as well as Glu368 from the SH3 domain. Both of these residues lie at the interface between the PDZ and SH3 domains.

## Supplementary Material

PDB reference: PDZ–peptide complex, 4uu5

PDB reference: ligand-free PDZ domain, 4uu6

Supporting Information.. DOI: 10.1107/S139900471402776X/mh5164sup1.pdf

## Figures and Tables

**Table 1:** X-ray data-collection and refinement statistics

	Pals1^PDZ^Crb1^ICD^ complex	Ligand-free Pals1^PDZ^
Data collection		
Space group	*P*4_1_22	*P*4_3_2_1_2
Unit-cell parameters ()	*a* = *b* = 74.7, *c* = 42.7	*a* = *b* = 43.1, *c* = 89.6
Resolution ()	52.841.23 (1.261.23)	39.591.80 (1.851.80)
Completeness (%)	99.7 (98.7)	99.7 (99.2)
Multiplicity	6.2 (5.3)	6.8 (6.9)
*R*_p.i.m._	0.018 (0.465)	0.026 (0.543)
*I*/(*I*)	17.7 (1.9)	5.7 (1.3)
Total No. of observations	220391 (13452)	59679 (4355)
No. of unique reflections	35570 (2559)	8766 (630)
CC_1/2_	0.999 (0.723)	0.999 (0.605)
Structure refinement		
Copies in asymmetric unit	1	1
*R*_work_ (%)	16.80	20.39
*R*_free_ (%)	18.32	22.35
No. of protein atoms	782	633
No. of ligand atoms	129	0
No. of solvent atoms	178	43
Mean *B* factor (^2^)	22.19	43.2
R.m.s.d., bonds ()	0.015	0.002
R.m.s.d., angles ()	1.936	0.638
Ramachandran plot (%)		
Favoured	93.4	97.6
Allowed	5.0	2.4
Outliers	1.6	0
