# Supplementary material for: Structures of the human Pals1 PDZ domain with and without ligand suggest gated access of Crb to the PDZ peptide-binding groove
Source: Acta Crystallogr D Biol Crystallogr. 2015 Feb 26;71(Pt 3):555–64. doi: 10.1107/S139900471402776X (PMC4356366; doi:10.1107/S139900471402776X)
Supplement: Supplementary file 1 [file d-71-00555-sup1.pdf]

BIOLOGICAL CRYSTALLOGRAPHY

Volume 71 (2015)

Supporting information for article:

Structures of human Pals1 PDZ domain with and without ligand suggest a gated access of Crb to the PDZ peptide-binding groove

Marina E. Ivanova, Georgina C. Fletcher, Nicola O'Reilly, Andrew G. Purkiss, Barry J. Thompson and Neil Q. McDonald

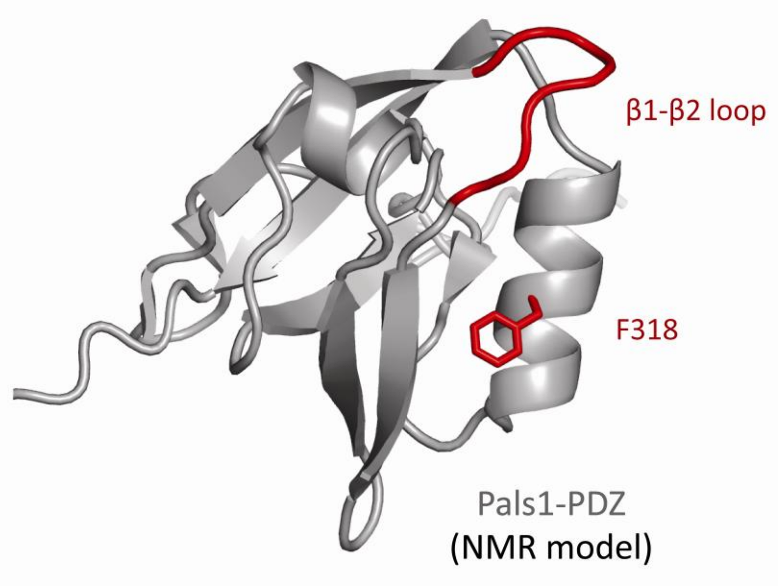

Figure S1 Phe318 rotamer and ß1-ß2 loop conformation from an NMR structure of Pals1^{PDZ} closely matches that of the apoPals1^{PDZ} x-ray structure Cartoon schematic of Pals1^{PDZ} NMR model. Pals1^{PDZ} is shown in grey; Pals1$^{Phe\text{-}318}$, with the same rotamer as the ligand free crystal structure and carboxylate-binding loop are shown in red.
